# The Sialic Acid Binding Activity of Human Parainfluenza Virus 3 and Mumps Virus Glycoproteins Enhances the Adherence of Group B Streptococci to HEp-2 Cells

**DOI:** 10.3389/fcimb.2018.00280

**Published:** 2018-08-17

**Authors:** Jie Tong, Yuguang Fu, Fandan Meng, Nadine Krüger, Peter Valentin-Weigand, Georg Herrler

**Affiliations:** ^1^Institute of Virology, University of Veterinary Medicine Hannover, Hanover, Germany; ^2^State Key Laboratory of Veterinary Biotechnology, Harbin Veterinary Research Institute, Chinese Academy of Agricultural Sciences, Harbin, China; ^3^Institute of Microbiology, University of Veterinary Medicine Hannover, Hanover, Germany

**Keywords:** sialic acids, hemagglutinin-neuraminidase protein, parainfluenza virus, mumps virus, group B streptococci, co-infection

## Abstract

In the complex microenvironment of the human respiratory tract, different kinds of microorganisms may synergistically interact with each other resulting in viral-bacterial co-infections that are often associated with more severe diseases than the respective mono-infections. Human respiratory paramyxoviruses, for example parainfluenza virus type 3 (HPIV3), are common causes of respiratory diseases both in infants and a subset of adults. HPIV3 recognizes sialic acid (SA)-containing receptors on host cells. In contrast to human influenza viruses which have a preference for α2,6-linked sialic acid, HPIV3 preferentially recognize α2,3-linked sialic acids. Group B streptococci (GBS) are colonizers in the human respiratory tract. They contain a capsular polysaccharide with terminal sialic acid residues in an α2,3-linkage. In the present study, we report that HPIV3 can recognize the α2,3-linked sialic acids present on GBS. The interaction was evident not only by the binding of virions to GBS in a co-sedimentation assay, but also in the GBS binding to HPIV3-infected cells. While co-infection by GBS and HPIV3 had a delaying effect on the virus replication, it enhanced GBS adherence to virus-infected cells. To show that other human paramyxoviruses are also able to recognize the capsular sialic acid of GBS we demonstrate that GBS attaches in a sialic acid-dependent way to transfected BHK cells expressing the HN protein of mumps virus (MuV) on their surface. Overall, our results reveal a new type of synergism in the co-infection by respiratory pathogens, which is based on the recognition of α2,3-linked sialic acids. This interaction between human paramyxoviruses and GBS enhances the bacterial adherence to airway cells and thus may result in more severe disease.

## Introduction

Human parainfluenza viruses (HPIV) were first isolated in the late 1950s from children sick with lower respiratory diseases. The virus differed from influenza viruses of the family *Orthomyxoviridae* by a non-segmented genome (Numazaki et al., 1968) and therefore, was assigned to a different virus family, *Paramyxoviridae*. (Burton, 1964; Cooney et al., 1975), HPIV3 causes acute respiratory infections (ARI) worldwide that are associated with a mortality of nearly 20% in children under 5 years-old (Glezen et al., 1984). HPIV has become one of the most threatening childhood infectious agents around the world due to insufficient vaccines or treatments (Counihan et al., 2001; Moscona, 2005; Garg et al., 2017).

HPIV3 and some other viruses of the family *Paramyxoviridae* share the common feature of recognizing sialic acid (SA)-containing receptors on host cells (Suzuki et al., 2001, 2004). Two surface glycoproteins, the hemagglutinin-neuraminidase (HN) protein and the fusion (F) protein, are involved in the initial steps of viral-cell interactions (Moscona and Peluso, 1992; Porotto et al., 2007). For optimal infection conditions, a balanced interaction between the sialic acid binding activity and the neuraminidase activity of the HN protein is essential (Tappert et al., 2013). HPIV3, the clinically most prevalent HPIV subtype, recognizes α2,3-linked sialic acids in branched and unbranched oligosaccharides present on either glycoproteins or glycolipids (Suzuki et al., 2001). Most efficient binding was observed with a sialylated tetrasaccharide (Amonsen et al., 2007). Mumps virus (MuV), another paramyxovirus, has recently been reported to use a trisaccharide containing α2,3-linked sialic acid in unbranched sugar chains as a receptor determinant (Kubota et al., 2016). Some strains especially neurovirulent variants may show an increased binding activity for α2,6-linked sialic acid (Reyes-Leyva et al., 2007). With respect to the sialic acid binding activity, the human paramyxoviruses HPIV3 and MuV differ from human influenza viruses which have a clear preference for the α2,6 linkage (Rogers and Paulson, 1983).

In the complex microenvironment of the human respiratory tract, different kinds of microorganisms may synergistically interact with each other resulting in viral-bacterial co-infections that are often associated with more severe disease than the respective mono-infections (Beadling and Slifka, 2004; Franz et al., 2010). Apart from influenza A viruses, paramyxoviruses including HPIV also have been frequently implicated in the pathogenesis of bacterial pneumonia in humans (Korppi et al., 1990; Juvén et al., 2000). Among the bacterial pathogens involved in respiratory co-infections, streptococci play a prominent role and *S. pneumonia* being a well-known representative. Group B streptococci (GBS, *Streptococcus agalactiae*), the causative agent of infant meningitis and pneumonia in non-pregnant adults have been found to colonize the human airway epithelium (Fallon and Sonnenwirth, 1978; Christensen et al., 1983; Doran et al., 2002). In a previous study, we demonstrated that GBS is able to interact with influenza viruses which have a preference for avian type receptors (α2,3-linked sialic acids). Streptococcal binding to the influenza hemagglutinin expressed on the surface of virus-infected cells enhances the bacterial adhesion and invasion properties (Tong et al., 2018). However, avian influenza viruses only occasionally infect humans (Stevens et al., 2006; Shi et al., 2013). In contrast to human influenza viruses which have a preference for α2,6-linked sialic acid, human paramyxoviruses recognize α2,3-linked sialic acid. Therefore we analyzed the interactions between GBS and a human paramyxovirus, HPIV3.

## Materials and methods

### Cells, viruses, bacteria and plasmids

Human laryngeal carcinoma epithelial cells (HEp-2, ATCC® CCL-23™) were maintained in MEM Eagle's Medium (EMEM, CytoGen AG, Germany) supplemented with 10% fetal calf serum (FCS, Millipore AG, Germany) and 2 mM stable L-glutamine. BHK-21 cells were grown in Dulbecco's minimum essential medium (DMEM; Gibco) supplemented with 5% FCS. Both cell lines were cultivated in 75-cm^2^ tissue culture flasks (Greiner Bio-One, Germany) at 37°C and 5% CO_2_.

Human parainfluenza virus type 3 (HPIV3, ATCC® VR-93, kindly provided by Albert Heim, Hannover Medical School) were propagated on HEp-2 cells and the viral titers were determined by plaque assay as described previously (Shibuta et al., 1981).

The virulent *Streptococcus suis* (*S. suis*) serotype 2 strain 10 and the clinical isolate of group B streptococcus (GBS) serotype III strain NEM316 were kindly provided by Hilde Smith (Wageningen Bioveterinary Research, Lelystad, The Netherlands) and Marcus Fulde (Institute for Microbiology and Epizootics, FU Berlin), respectively. For all infection experiments, cryo-conserved bacterial stocks were used and prepared as described previously (Willenborg et al., 2014).

The open reading frame of MuV-HN (GenBank accession no.: KM519600) was cloned into the expression vector pCG1 and connected with a sequence coding for a FLAG epitope (DYKDDDDK) at the C-terminal end (Krüger et al., 2015).

### Neuraminidase treatment

For desialylation, bacteria cultures or virus-bacteria mixtures were incubated with 200 mU or 500 mU neuraminidase (NA) type V from *Clostridium perfringens* (Sigma Aldrich, Munich) for 1 h at 37°C, then the enzyme was removed and bacteria were washed three times with cold PBS before subjected to co-sedimentation assays or co-infection of HEp-2 cells, respectively.

### Co-sedimentation of HPIV3 and streptococci

Co-sedimentation assays were performed as described previously (Tong et al., 2018). Suspensions containing both HPIV3 (1 × 10^5^ PFU/ml) and bacteria (1 × 10^8^ CFU) were incubated on an overhead shaker for 1 h at 4°C and subsequently subjected to low-speed centrifugation at 6,000 rpm for 10 min to pellet bacteria but not free virus. The supernatants were analyzed for the presence of virus by determining (i) the HA activity with 1% guinea pig erythrocytes (gpRBC, Dune Lab, Germany) and (ii) the infectivity by virus titration on HEp-2 cells. The bacteria pellets were washed three times with cold PBS containing antibiotics (200 U/ml penicillin/streptomycin) before determining the infectivity by plaque assays on HEp-2 cells. To measure virus elution, the bacteria pellets were re-suspended by PBS with or without NA and incubated for 1 h at 37°C. At different time points (0, 20, 40, and 60 min) aliquots were collected for HA titration.

### Co-infection of HEp-2 cells by HPIV3 and streptococci

HEp-2 cells were grown in 24-well plates. For evaluation of viral replication kinetics, cells were inoculated with HPIV3 at a multiplicity of infection (MOI) of 0.5 for 2 h at 37°C. After three washing steps with PBS, *S. suis* (MOI = 100) or GBS (MOI = 125) were added to the cells and maintained at 37°C for another 2 h. Then the cells were washed thoroughly with PBS and further incubated in EMEM containing 2% FCS. At 8, 24, 48, 72, 96 h post virus infection (p.i.), the supernatants were collected and virus titration was performed on HEp-2 cells as previously described (Shibuta et al., 1981).

To analyze bacterial adherence by microscopy, the medium was removed from virus-infected cells at 16 h p.i. and washed thoroughly with PBS, followed by inoculation with *S. suis* (MOI = 100) or GBS (MOI = 125) for 1 h at room temperature or at 4°C, respectively. Finally, the co-infected cells were washed and fixed with 3% paraformaldehyde and further subjected to immunofluorescence microscopy. To determine the importance of sialic acids on the bacterial capsular polysaccharide, bacteria were pretreated with NA as described above.

### Determination of total cell-associated bacteria

The total cell-associated bacteria were calculated as previously described (Tong et al., 2018), but without antibiotic treatment of the infected cells. Briefly, at 16 h after HIPV3-infection cells were incubated with bacteria. Then the cells were lysed and serial dilutions of the lysates in PBS were plated on Columbia agar supplemented with 7% sheep blood to determine colony forming units (CFU) indicating the number of total cell-associated bacteria, i.e., adherent and internalized bacteria.

### GBS binding to MuV-HN expressed on BHK-21 cells

BHK-21 cells were seeded on cover slips and transfection with MuV-HN expression plasmids was performed using the Lipofectamine 2000® reagent (Invitrogen, Germany) according to the manufacturer's protocol. At 1 day post transfection (p.t.), the cells were washed with PBS and inoculated with GBS (MOI = 125) or *S. suis* (MOI = 100) for 1 h at 4°C prior to fixation for IFA. Cells transfected with the empty vector pCG1 were used as controls. Three independent experiments were performed as duplicates.

### Hemadsorption assays

To investigate the hemadsorption activity of MuV-HN, BHK-21 cells were grown in 24 well plates and transfected for the expression of MuV-HN. At 1 day p.t., cells were washed with PBS. One well was fixed and subjected to immunofluorescence microscopy to investigate the MuV-HN expression level. Three additional wells of transfected cells were inoculated with GBS (MOI = 125), *S. suis* (MOI = 100) or PBS for 30 min at room temperature. After removing the bacteria by washing with PBS, cells were further incubated with 1% guinea pig red blood cells for 30 min at 4°C or room temperature. Next, cold distilled water was added to lyse the adherent erythrocytes on the surface of the BHK-21 cells. The amounts of released hemoglobin were estimated by determining the OD value at 410 nm. Three independent experiments were performed.

### Immunofluorescence analysis (IFA)

Immunostaining of the cells was performed as described previously (Charland et al., 1996; Liang et al., 2008). For the detection of HPIV3, a virus-specific anti-serum (goat, VMR Int.) was used followed by an Alexa Fluor® 568-conjugated secondary antibody raised against goat IgG (H+L) (Invitrogen, Germany). For visualization of bacteria, rabbit anti-*S. suis* and rabbit anti-GBS antisera were used as primary antibodies followed by Alexa Fluor® 488-conjugated anti-rabbit IgG (H+L) antibodies (Invitrogen). MuV-HN was detected by an anti-mouse FLAG-M2 antibody (Sigma-Aldrich, Germany) and Alexa Fluor® 568-conjugated secondary antibodies directed against anti-mouse IgG (H+L) antibody (Invitrogen, Germany).

## Statistical analysis

For statistical analysis in hemadsorption assays, two-tailed, unpaired *t*-tests were used. One-way ANOVA analyses were performed in other parts of the experiments using the GraphPad Prism 5 software (Prism 5 for Windows; GraphPad Software, San Diego, CA, USA).

## Results

### Sialic acid-dependent interactions between HPIV3 and GBS

**Figure 1:**
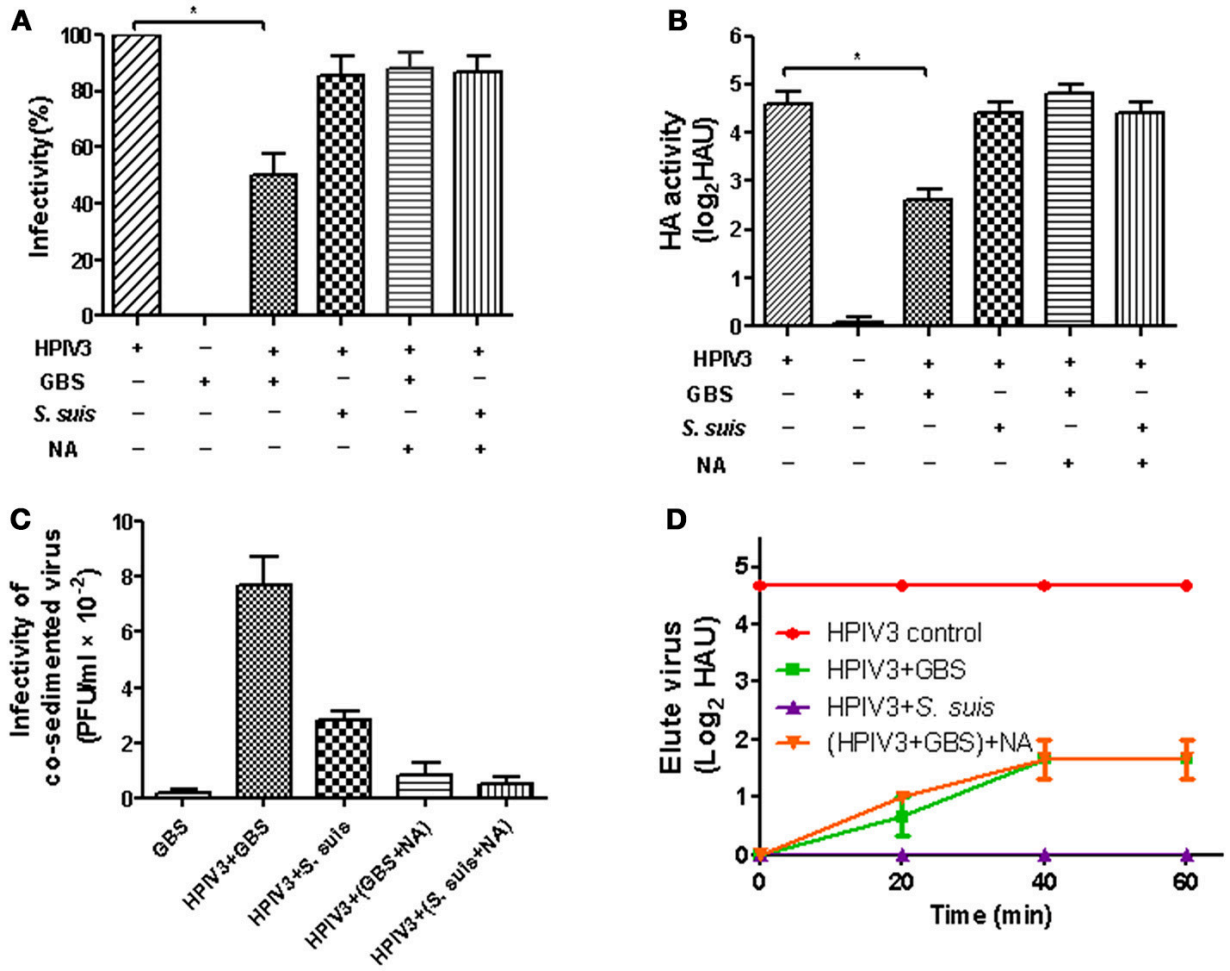
HA activity and infectivity of HPIV3 after co-sedimention with GBS or *S. suis*. After incubation of HPIV3 with GBS, NA-pretreated GBS or *S. suis*, the bacteria were pelleted by low-speed centrifugation. The supernatants were analyzed for infectivity **(A)** by plaque titration on HEp-2 cells or for HA activity **(B)** using guinea pig erythrocytes. The pellet fractions **(C)** were analyzed for the presence of infectious viruses by plaque titration on HEp-2 cells. In **(D)**, pelleted bacteria were resuspended in PBS with or without NA and incubated for 1 h at 37°C. At the times indicated, aliquots were collected for HA titration to detect the eluted virus. Statistical significance was determined with one-way ANOVA, *P<*0.05*.

We have recently shown that the α2,3-linked sialic acids on the capsular polysaccharide of GBS are recognized by several avian influenza viruses but not by human and porcine influenza viruses that have a preference for the α2,6-linkage type (Cundell and Tuomanen, 1994; Strohal et al., 1994; Tong et al., 2018). In contrast to human influenza viruses, HPIV3 utilizes α2,3-linked sialic acid as a receptor determinant for binding to and infection of cells (Suzuki et al., 2001). Therefore, we were interested whether this virus is able to interact with encapsulated GBS in a sialic acid-dependent manner. In a co-sedimentation experiment, HPIV3 was incubated for 1 h with either GBS or, as a control, with *S. suis*. The latter bacterium contains a similar capsular polysaccharide as does GBS, but differs in the terminal sialic acid residue which is attached in an α2,6 rather than in an α2,3-linkage (Charland et al., 1996; Van Calsteren et al., 2010). Bacteria were sedimented by low-speed centrifugation and the supernatants were analyzed for the presence of virus. The infectivity titer was not affected by the presence of *S. suis*. However, a reduction by about 50% was determined for the sample containing GBS (Figure 1A). The amount of virus remaining in the supernatant could not be further decreased by increasing the amount of bacteria (Supplemental Figure 1). When the presence of virus was analyzed by HA titration, a four-fold reduction was obtained for the sample containing GBS (Figure 1B). It is not clear why a substantial amount of virus remained in the supernatant. Possible reasons may be related: (i) to the centrifugation procedure, (ii) to the presence of a viral subpopulation that recognizes the capsular sialic acid only with low affinity, (iii) to the fact that in the case of paramyxoviruses —in contrast to influenza viruses - the sialic acid binding and the neuraminidase activity are located on the same surface protein (HN) which may make it more difficult to control the sialidase activity; or (iv) to a combination of the above reasons. To get direct evidence for the presence of virus in the pellet fraction, the sediment was suspended and subjected to plaque assays. The highest infectivity titer was found in the pellet fraction obtained after co-sedimentation of HPIV3 and GBS (Figure 1C). We further analyzed whether bound virus can elute from GBS. The GBS-HPIV3 mixtures were incubated at 37°C, followed by sedimentation of bacteria by low-speed centrifugation. After suspension of the sedimented bacteria, HA titration indicated that a substantial amount of virus had eluted from bacteria after 40 min (Figure 1D). The viral neuraminidase activity of the HN protein was sufficient for this effect (green line, indicated as HPIV+GBS), because elution could not be enhanced by addition of exogenous bacterial neuraminidase [orange line, indicated as (HPIV+GBS) + NA].

### Adherence of GBS to respiratory epithelial cells is enhanced after HPIV3 infection

**Figure 2:**
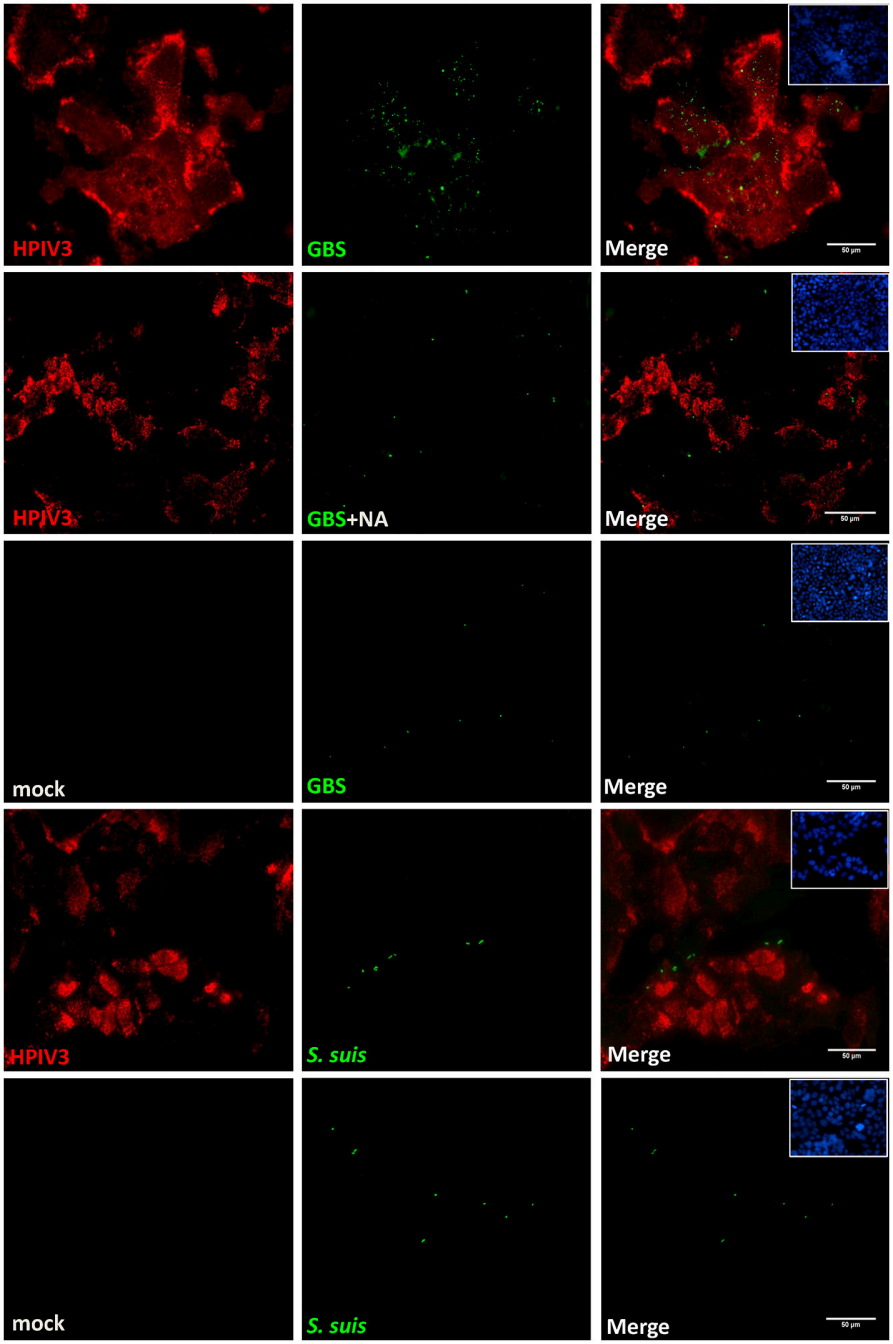
Immunofluorescence analysis of streptococcal attachment to HPIV3-infected cells. HEp-2 cells infected by HPIV3 were inoculated at 16 h.p.i. with GBS or *S. suis* or neuraminidase-treated GBS (GBS+NA) and incubated for 1 h at 4°C. Bound bacteria (green) and HPIV-infected cells (red) were immune-stained with specific polyclonal antibodies. All experiments were repeated at least three times with duplicate samples. Scale bars represent 50 μm.

**Figure 3:**
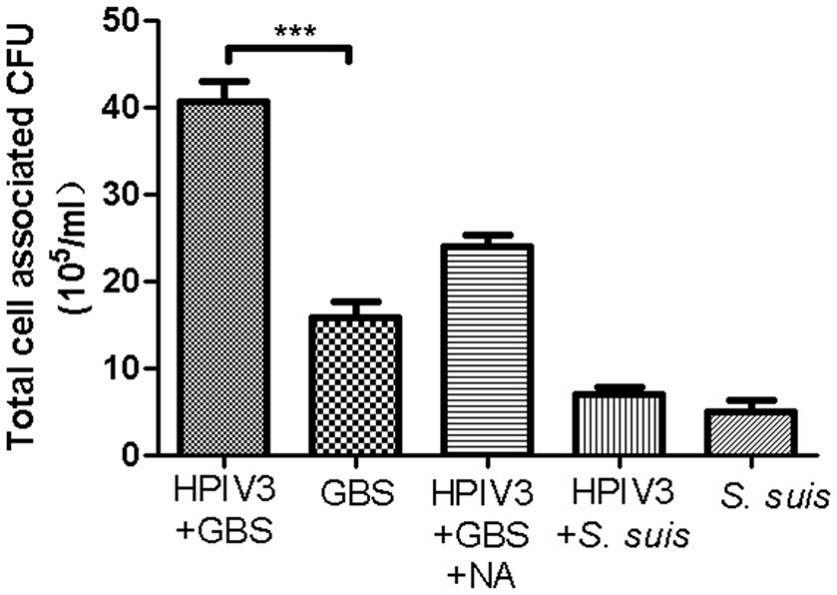
Quantification of bacteria adhering to HPIV-infected HEp-2 cells. GBS (with or without NA-treatment) or *S. suis* were inoculated onto HEp-2 cells at 16 h post infection by HPIV. Parallel experiments were performed on HEp-2 cells without virus infection. Total cell-associated bacteria were determined by serial plating on Columbia agar supplemented with 7% sheep blood. All experiments were repeated at least three times with duplicate samples. Statistical analysis was performed with one-way ANOVA, ***P<*0.001*.

To analyze the viral-bacterial interactions during co-infections, HEp-2 cells were pre-infected by HPIV3, and at 16 h post-virus infection, they were inoculated with either GBS or *S. suis*. After an adherence period of 1 h at 4°C, cells were analyzed by immunofluorescence microscopy. As shown in Figure 2, most fluorescent signals for bound bacteria (green) were found in the sample co-infected by HPIV3 and GBS, and all adherent bacteria were associated with virus-infected cells (red). When co-infection was performed with either *S. suis* or with neuraminidase-treated GBS, the number of fluorescent signals was low and not strongly associated with virus-infected cells (Figure 2). For quantification of cell-associated bacteria, cells were lysed and the colony-forming units were determined. Compared to GBS-mono-infection, a 2.5-fold increased number of bacteria were associated with cells co-infected by HPIV3 (Figure 3). Notably, these numbers include both adherent and internalized bacteria. Desialylation of bacteria by prior neuraminidase-treatment reduced the number of bound bacteria to a value close to that determined for the GBS-mono-infected sample. *S. suis* was less efficient in binding to cells than was GBS, and the number of cell-associated bacteria was not significantly increased when cells were pre-infected by HPIV3 (Figure 3). Thus, HPIV3 infection enhanced the adherence of GBS to HEp-2 cells in a sialic acid-dependent manner.

### Co-infection by GBS delays the replication of HPIV3

**Figure 4:**
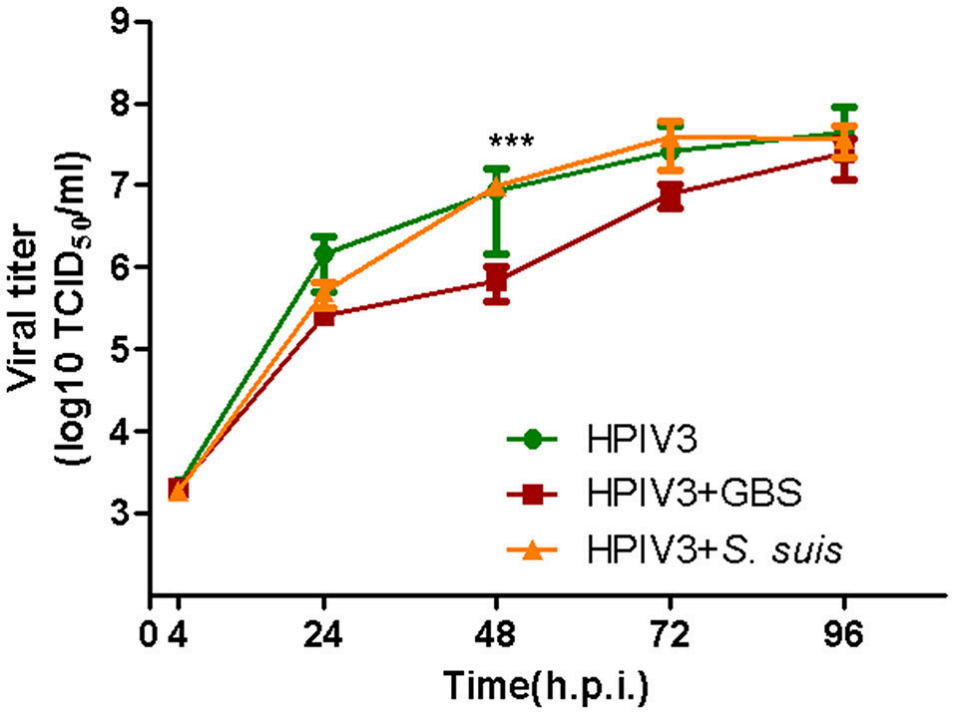
Effects of co-infection by streptococci on the replication kinetics of HPIV in HEp-2 cells. HEp−2 cells were infected by HPIV (MOI = 0.5), followed by 2 h incubation with GBS (red line) or *S. suis* (orange line). Supernatants were harvested at different time point post-virus infection and titrated by determining the TCID_50_ on HEp-2 cells. Results represent the mean value of virus titers ± SEM pooled from three independent experiments with duplicated samples. Asterisks indicate significant differences at 48 h.p.i. between cells infected by virus alone (green lines) and cells co-infected by HPIV and GBS (red line). Statistical analysis was performed with one-way ANOVA, ***P<*0.001*.

Having shown that HPIV3-GBS co-infection enhances the bacterial adherence, we also analyzed whether it affects viral replication. At 2 h post virus-infection, cells were incubated for another 2 h at 37°C with either GBS or *S. suis*. After removal of non-adherent bacteria, cells were further incubated and at different time points aliquots were taken for virus titration. At 48 h post virus-infection, cells co-infected by GBS showed one-fold significantly lower infectivity titer compared to cells infected by virus only (Figure 4). At 72 h.p.i., the difference was less pronounced and at 96 h.p.i., both samples showed a similar titer. Co-infection with *S. suis* had no effect on the replication kinetics of HPIV3 (Figure 4, orange line). Thus, whereas adherence of GBS is enhanced by co-infection with HPIV3, viral replication is delayed under co-infection conditions.

### GBS bind to MuV HN protein expressed on BHK-21 cells and impede the hemadsorption activity of HN-expressing cells

**Figure 5:**
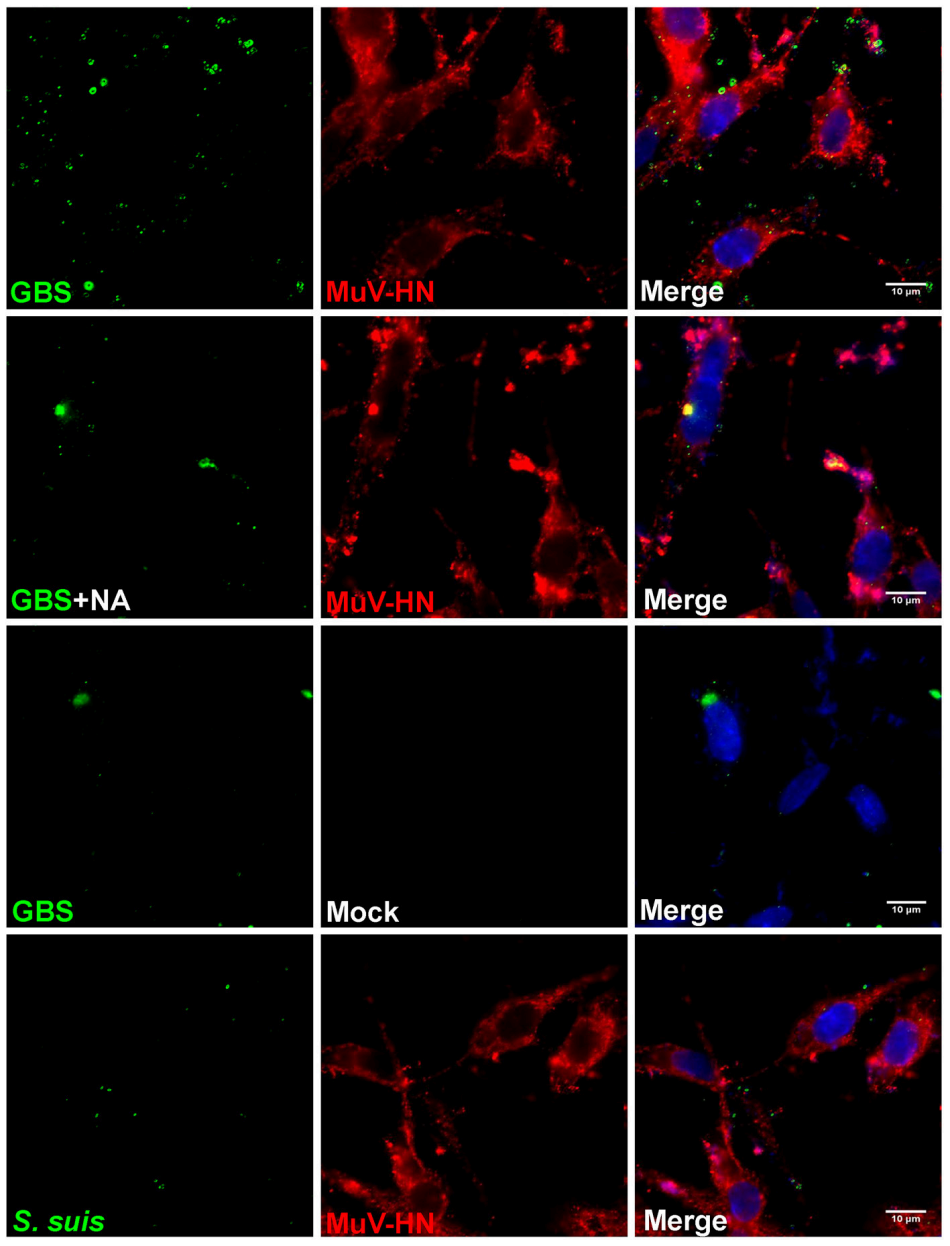
Immunofluorescence analysis of streptococcal attachment to HN-expressing cells. HEp-2 cells were transfected for expression of MuV-HN protein. At 24 h p.t., GBS (with or without NA-treatment) or *S. suis* were inoculated onto the cells at 4°C for 1 h. Cells expressing no MuV-HN protein are indicated as “mock.” After adherence of bacteria, cells were immuno-stained for MuV-HN (red), bacteria (green), or nuclei (blue) and analyzed by fluorescence microscopy (scale bar: 10 μm).

**Figure 6:**
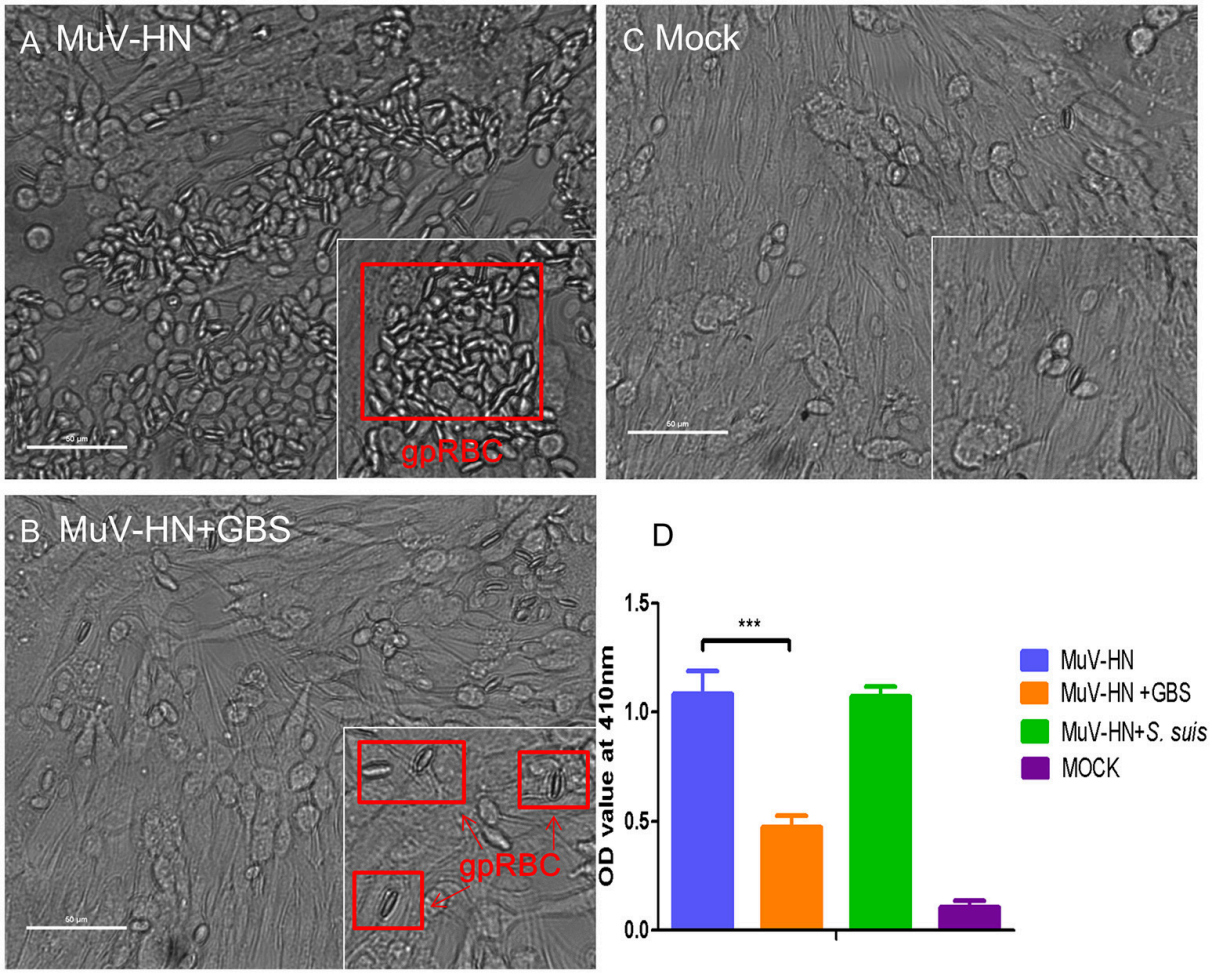
Hemadsorption of MuV-HN. HEp-2 cells were transfected for the expression of MuV-HN. At 24 h p.t., cells were incubated with or without streptococci (GBS or *S. suis*) for 30 min followed by 15 min incubation with 2% guinea pig red blood cells (gpRBC) at 4°C. Adhering red blood cells are highlighted by red boxes in **(A)** and **(B)**. Untransfected control cells (expressing no MuV-HN protein, designated “mock”) are shown in **(C)**. Adhering erythrocytes were lysed and the OD values of the supernatants at 410 nm were measured for evaluating the amount of hemoglobin in **(D)**. As *S. suis* had no inhibitory effect on the hemadsorption activity of MuV-HN, the result is shown only in the bar chart **(D)**. All experiments were repeated at least three times with duplicate samples. Statistical analysis was performed with unpaired Student's *t*-test, ***P<*0.001*. Scale bars in **(A)**, **(B)**, and **(C)** represent 50 μm.

Like HPIV3, MuV has been reported to use α2,3-linked sialic acid as a receptor determinant for binding to and infection of target cells (Kubota et al., 2016). To analyze whether the glycoproteins of MuV can recognize the capsular sialic acid of GBS, BHK-21 cells were transfected for expression of the MuV-HN protein on the cell surface. Bacterial adherence was determined by incubation with GBS for 1 h at 4°C. As shown in Figure 5 (top panels), a large number of green fluorescent signals indicated the presence of GBS adhering to MuV-HN expressing cells. The adherence of the bacteria was dependent on the α2,3-linked sialic acids in the capsular polysaccharide, because a much lower number of fluorescent signals was detected when GBS had been pretreated with neuraminidase or when GBS was replaced by *S. suis* (Figure 5) which contains α2,6-linked sialic acids in its capsular polysaccharide. To get further evidence for the binding of the MuV-HN protein to GBS, we performed a hemadsorption assay. Consistent with previous reports that MuV agglutinates guinea pig erythrocytes, these red blood cells specifically attached to HN-expressing BHK-21 cells (compare Figures 6A,C). In the presence of GBS, adherence of the erythrocytes was substantially reduced (Figures 6B,D). No inhibitory effect was detected when *S. suis* was applied (shown only in the bar chart, Figure 6D). These results confirm that the MuV glycoproteins interact with GBS in a manner that is dependent on the α2,3-linked sialic acids of the capsular polysaccharide. This method can also be applied to the glycoproteins of other paramyxoviruses that are less well characterized. When there is an indication that infection by the respective virus is enhanced by GBS, the analysis has to proceed from the stage of transfected cells to the stage of virus-infected cells.

## Discussion

Many influenza and paramyxoviruses use sialic acid as a receptor determinant for binding to and initiating infection of target cells. The binding of the respective viral proteins to the sugar alone, N-acetylneuraminic acid, is characterized by a low affinity. The binding to the cell surface receptors occurs with higher affinity because viruses recognize the terminal sialic acid as part of the larger oligosaccharide motif present on glycoproteins or glycolipids. The interaction with viral proteins is dependent not only on the type of sialic acid but also on other factors, e.g., the linkage to the neighboring sugar which most often is galactose (Matrosovich et al., 2015). Influenza viruses are well-known for their differential interaction with sialic acid; thus, avian viruses recognize α2,3-linked sialic acid and human viruses prefer α2,6-linked sialic acid (Rogers and Paulson, 1983; Connor et al., 1994). This binding specificity is not observed on human paramyxoviruses (Moscona and Peluso, 1996). Most members of the genera *Respirovirus*, including HPIV3, and *Rubulavirus*, including MuV, appear to attach to glycoconjugates containing α2,3-linked sialic acid, though some strains/variants have been reported to recognize α2,6-linked sialic acid (Reyes-Leyva et al., 2007; Kubota et al., 2016). Whereas the recognition of α2,3-linked sialic acid is a common feature of human paramyxoviruses, differences have been reported with respect to the neighboring sugars. The binding affinity may depend on whether the glycan is branched or unbranched, or whether the following sugars are modified by sulfate groups or fucose residues (Amonsen et al., 2007). In this way the viruses may interact with a broader or with a narrower spectrum of glycan structures, and therefore they may also differ in the spectrum of target cells.

Because of the considerations mentioned above, it was not possible to predict whether HPIV3 and MuV glycoproteins are able to interact with GBS. The capsular polysaccharide of this microorganism contains a rhamnose residue that is not found in glycoconjugates of vertebrate cells. Glycan arrays used to characterize the binding activity of viral proteins usually do not include glycans with a rhamnose. Our results demonstrate that the rhamnose residue does not affect the conformation of the respective glycan structure in a way that prevents the recognition by either HPIV3 or MuV. In fact, the interaction of the two paramyxoviruses with GBS occurs with the same sialic acid specificity as does the interplay between these viruses and their host cells. Viral-bacterial co-sedimentation or bacterial adherence to HN-expressing cells was observed only with GBS which contains α2,3-linked sialic acid, but not with *S. suis* which has a similar capsular polysaccharide but differs in the terminal sialic acid which is present in an α2,6-linkage (Van Calsteren et al., 2010).

The binding of the paramyxoviruses to GBS appears to occur with similar affinity as does the interaction with cell surface receptors. This conclusion is based on our finding that GBS is able to inhibit the hemadsorption activity of the MuV-HN protein competitively. Efficient attachment of influenza and paramyxoviruses to the cell surface depends not only on the specific recognition of cellular receptors, but also on the establishment of multiple binding sites. Multivalent interactions between viruses and cells can be achieved because a virion contains hundreds of surface glycoproteins and cells usually expose a large number of sialylated glycan motifs on their surface. In our case, multivalent interactions can also be achieved with GBS because this microorganism contains many α2,3-linked sialic acids in the capsule.

Recently we reported that co-infection of cells by avian influenza viruses and GBS results in a delay of the viral replication (Tong et al., 2018). A similar result was obtained in the present study with HPIV3. This negative effect may be due to binding of bacteria to virus infected-cells which may delay the budding process and virus egress. An alternative explanation is that binding of bacteria to virions released from infected cells may impede virus entry into uninfected cells and thus delay the spread of infection.

Whereas viral-bacterial co-infection had a negative effect on the replication of HPIV3, adherence of GBS was greatly enhanced. Several components of GBS have been described to mediate the adherence to host factors (for a review see Wang et al., 2015). Most important for binding to and colonization of epithelial cells appear to be the adhesins BsaB, HvgA, the Alpha C proteins (ACP), the serine-rich repeat (Srr) proteins as well as the pili. We do not know which of these proteins is involved in the adherence of GBS to HEp-2 cells, but our results show that sialic acid-mediated binding to HN proteins exposed on the cell surface is a more efficient way of adherence. Previously, sialic acids of GBS have mainly been associated with immune evasion by interaction with lectins on immune cells which prevents complement activation and counteracts the bactericidal activity of the host cells (Chang et al., 2014; Landwehr-Kenzel and Henneke, 2014). Our results demonstrate that the capsular sialic acids of GBS enhance the adherence to paramyxovirus-infected cells which is consistent with our recent report that the HA proteins of avian influenza viruses expressed on infected cells enhance the adhesion properties of GBS. As shown with influenza virus-infected cells, enhanced adherence is paralleled by enhanced invasion. GBS is a commensal bacterium that asymptomatically colonizes mucosal surfaces. To cause disease, the microorganism has to invade the host. Therefore, via increased adherence and invasion, co-infection with influenza viruses or paramyxoviruses that recognize α2,3-linked sialic acid may contribute to the severity of disease caused by GBS. It will be interesting in the future, to see how co-infection by GBS and HPIV3 contributes to viral/bacterial replication and pathogenesis in a small animal model.

Mumps virus infections are limited in developed countries because of vaccination programmes. Therefore, information about bacterial co-infections is lacking. However, they may play a role in developing countries. Furthermore, our results show that sialic acid-dependent interactions with GBS are not limited to HPIV3 but can occur also with other viruses, and this type of interaction can also be demonstrated by a different method, i.e., with transfected cells. The latter approach may be interesting for viruses that are difficult to amplify in cell cultures.

Another streptococcal partner for viral bacterial co-infections is *S. pneumoniae*. This pathogen contains a neuraminidase to release sialic acid from the cell surface and therefore does not contain sialic acid residues in the capsular polysaccharide. In co-infections with influenza viruses, it may also use the viral neuraminidase for desialylation purposes. It will be interesting in the future to compare the strategies of GBS and *S. pneumoniae* to increase the adherence and invasion, the sialic acid-dependent interaction of GBS and the neuraminidase-based strategy of *S. pneumoniae*.

## Author contributions

GH and PV-W conceived and designed the study. JT performed the experiments. JT, FM, YF, NK analyzed the data. JT, GH, FM, PV-W, NK wrote the paper. All authors read and approved the final manuscript.

### Conflict of interest statement

The authors declare that the research was conducted in the absence of any commercial or financial relationships that could be construed as a potential conflict of interest.

## Supplementary material

Supplemental Figure 1 Co-sedimention of HPIV3 with GBS or *S. suis* at different virus/bacteria ratios. Bacteria (10^8^ CFU) were mixed with different amounts of infectious virus. After incubation of HPIV3 with GBS, NA-pretreated GBS or *S. suis*, the bacteria were pelleted by low-speed centrifugation. The supernatants were analyzed for viral infectivity by plaque titration on HEp-2 cells. Statistical significance was determined with one-way ANOVA, ^*^*P* < 0.05. ns, not significant.

## References

[B1] Amonsen M. Smith D. F. Cummings R. D. Air G. M. (2007). Human parainfluenza viruses hPIV1 and hPIV3 bind oligosaccharides with alpha 2-3-linked sialic acids that are distinct from those bound by H5 avian influenza virus hemagglutinin. J. Virol. 81, 8341–8345. 10.1128/JVI.00718-07 17522226 PMC1951310

[B2] Beadling C. Slifka M. K. (2004). How do viral infections predispose patients to bacterial infections? Curr. Opin. Infect. Dis. 17, 185–191. 10.1097/01.qco.0000129612.21745.08 15166819

[B3] Burton J. F. (1964). Respiratory syncytial virus and parainfluenza myxoviruses as causal agents of acute respiratory infections in auckland children. N. Z. Med J. 63, 372–374. 14171936

[B4] Chang Y. C. Olson J. Beasley F. C. Tung C. Zhang J. Crocker P. R.. (2014). Group B Streptococcus Engages an inhibitory siglec through sialic acid mimicry to blunt innate immune and inflammatory responses *in vivo*. Plos. Pathogens 10:e1003846. 10.1371/journal.ppat.1003846 24391502 PMC3879367

[B5] Charland N. Kobisch M. Martineau-Doizé B. Jacques M. Gottschalk M. (1996). Role of capsular sialic acid in virulence and resistance to phagocytosis of Streptococcus suis capsular type 2. Fems Immunol. Med. Microbiol. 14, 195–203. 10.1111/j.1574-695X.1996.tb00287.x 8856318

[B6] Christensen K. K. Christensen P. Dahlander K. Lindén V. Lindroth M. Svenningsen N. (1983). The significance of group B streptococci in neonatal pneumonia. Eur. J. Pediatr. 140, 118–122. 10.1007/BF00441657 6350005

[B7] Connor R. J. Kawaoka Y. Webster R. G. Paulson J. C. (1994). Receptor specificity in human, avian, and equine H2 and H3 influenza-virus isolates. Virology 205, 17–23. 10.1006/viro.1994.1615 7975212

[B8] Cooney M. K. Fox J. P. Hall C. E. (1975). The seattle virus watch. VI. observations of infections with and illness due to parainfluenza, mumps and respiratory syncytial viruses and mycoplasma pneumoniae. Am. J. Epidemiol. 101, 532–551. 168766 10.1093/oxfordjournals.aje.a112125

[B9] Counihan M. E. Shay D. K. Holman R. C. Lowther S. A. Anderson L. J. (2001). Human parainfluenza virus-associated hospitalizations among children less than five years of age in the United States. Pediatr. Infect. Dis. J. 20, 646–653. 10.1097/00006454-200107000-00003 11465835

[B10] Cundell D. R. Tuomanen E. I. (1994). Receptor specificity of adherence of streptococcus-pneumoniae to human type-ii pneumocytes and vascular endothelial-cells *in-vitro*. Microb. Pathog. 17, 361–374. 10.1006/mpat.1994.1082 7752878

[B11] Doran K. S. Chang J. C. Benoit V. M. Eckmann L. Nizet V. (2002). Group B streptococcal beta-hemolysin/cytolysin promotes invasion of human lung epithelial cells and the release of interleukin-8. J. Infect. Dis. 185, 196–203. 10.1086/338475 11807693

[B12] Fallon M. D. Sonnenwirth A. C. (1978). Occurrence of Group-B streptococcus in adults. Clin. Res. 26, A769–A769.

[B13] Franz A. Adams O. Willems R. Bonzel L. Neuhausen N. Schweizer-Krantz S.. (2010). Correlation of viral load of respiratory pathogens and co-infections with disease severity in children hospitalized for lower respiratory tract infection. J. Clin. Virol. 48, 239–245. 10.1016/j.jcv.2010.05.007 20646956 PMC7185496

[B14] Garg R. Brownlie R. Latimer L. Gerdts V. Potter A. van Drunen Littel-van den Hurk S. (2017). Vaccination with a human parainfluenza virus type 3 chimeric FHN glycoprotein formulated with a combination adjuvant induces protective immunity. Vaccine 35, 7139–7146. 10.1016/j.vaccine.2017.10.095 29153777

[B15] Glezen W. P. Frank A. L. Taber L. H. Kasel J. A. (1984). Parainfluenza virus type 3: seasonality and risk of infection and reinfection in young children. J. Infect. Dis. 150, 851–857. 10.1093/infdis/150.6.851 6094674

[B16] Juvén T. Mertsola J. Waris M. Leinonen M. Meurman O. Roivainen M.. (2000). Etiology of community-acquired pneumonia in 254 hospitalized children. Pediatr. Infect. Dis. J. 19, 293–298. 10.1097/00006454-200004000-00006 10783017

[B17] Korppi M. Leinonen M. Mäkelä P. H. Launiala K. (1990). Bacterial involvement in parainfluenza virus-infection in children. Scand. J. Infect. Dis. 22, 307–312. 10.3109/00365549009027052 2164707

[B18] Krüger N. Hoffmann M. Drexler J. F. Müller M. A. Corman V. M. Sauder C.. (2015). Functional properties and genetic relatedness of the fusion and hemagglutinin-neuraminidase proteins of a mumps virus-like bat virus. J. Virol. 89, 4539–4548. 10.1128/JVI.03693-14 25741010 PMC4442385

[B19] Kubota M. Takeuchi K. Watanabe S. Ohno S. Matsuoka R. Kohda D. (2016). Trisaccharide containing alpha 2,3-linked sialic acid is a receptor for mumps virus. Proc. Nat. Acad. Sci. U.S.A. 113, 11579–11584. 10.1073/pnas.1608383113 PMC5068328 27671656

[B20] Landwehr-Kenzel S. Henneke P. (2014). Interaction of streptococcus agalactiae and cellular innate immunity in colonization and disease. Front Immunol. 5:519. 10.3389/fimmu.2014.00519 25400631 PMC4212683

[B21] Liang G. Z. Chen Z. C. Yang S. B. Mei H. Zhou Y. Yang L. (2008). Recognition for avian influenza virus proteins based on support vector machine and linear discriminant analysis. Sci. China Ser. B Chem. 51, U166–U123. 10.1007/s11426-008-0006-7 PMC7089115 38624277

[B22] Matrosovich M. Herrler G. Klenk H. D. (2015). Sialic acid receptors of viruses. Top. Curr. Chem. 367, 1–28. 10.1007/128_2013_466 23873408 PMC7120183

[B23] Moscona A. (2005). Entry of parainfluenza virus into cells as a target for interrupting childhood respiratory disease. J. Clin. Investigat. 115, 1688–1698. 10.1172/JCI25669 16007245 PMC1159152

[B24] Moscona A. Peluso R. W. (1992). Fusion properties of cells infected with human parainfluenza virus type 3: receptor requirements for viral spread and virus-mediated membrane fusion. J. Virol. 66, 6280–6287. 1328668 10.1128/jvi.66.11.6280-6287.1992 PMC240119

[B25] Moscona A. Peluso R. W. (1996). Analysis of human parainfluenza virus 3 receptor binding variants: evidence for the use of a specific sialic acid-containing receptor. Microb. Pathog. 20, 179–184. 10.1006/mpat.1996.0016 8965678

[B26] Numazaki Y. Shigeta S. Ishida N. (1968). Studies on parainfluenza virus infections among infants and children in sendai .I. Isolation and identification methods of parainfluenza viruses. Jpn. J. Microbiol. B. 12, 275–281. 4303467 10.1111/j.1348-0421.1968.tb00396.x

[B27] Porotto M. Fornabaio M. Kellogg G. E. Moscona A. (2007). A second receptor binding site on human parainfluenza virus type 3 hemagglutinin-neuraminidase contributes to activation of the fusion mechanism. J. Virol. 81, 3216–3228. 10.1128/JVI.02617-06 17229690 PMC1866072

[B28] Reyes-Leyva J. Baños R. Borraz-Argüello M. Santos-López G. Rosas N. Alvarado G.. (2007). Amino acid change 335 E to K affects the sialic-acid-binding and neuraminidase activities of Urabe AM9 mumps virus hemagglutinin-neuraminidase glycoprotein. Microb. Infect. 9, 234–240. 10.1016/j.micinf.2006.11.011 17223599

[B29] Rogers G. N. Paulson J. C. (1983). Receptor determinants of human and animal influenza-virus isolates - differences in receptor specificity of the hemagglutinin-H-3 based on species of origin. Virology 127, 361–373. 10.1016/0042-6822(83)90150-2 6868370

[B30] Shi Y. Zhang W. Wang F. Qi J. Wu Y. Song H.. (2013). Structures and receptor binding of hemagglutinins from human-infecting H7N9 influenza viruses. Science 342, 243–247. 10.1126/science.1242917 24009358

[B31] Shibuta H. Kanda T. Hazama A. Adachi A. Matumoto M. (1981). Parainfluenza 3 virus: plaque-type variants lacking neuraminidase activity. Infect. Immun. 34, 262–267. 6271683 10.1128/iai.34.1.262-267.1981 PMC350851

[B32] Stevens J. Blixt O. Tumpey T. M. Taubenberger J. K. Paulson J. C. Wilson I. A. (2006). Structure and receptor specificity of the hemagglutinin from an H5N1 influenza virus. Science 312, 404–410. 10.1126/science.1124513 16543414

[B33] Strohal R. Paucz L. Pehamberger H. Stingl G. (1994). T-Cell receptor repertoire of lymphocytes infiltrating cutaneous melanoma is predominated by V-Alpha specificities present in T-Cells of normal human skin. Cancer Res. 54, 4734–4739. 8062272

[B34] Suzuki T. Portner A. Scroggs R. A. Uchikawa M. Koyama N. Matsuo K.. (2001). Receptor specificities of human respiroviruses. J. Virol. 75, 4604–4613. 10.1128/JVI.75.10.4604-4613.2001 11312330 PMC114213

[B35] Suzuki T. Takimoto T. Portner A. Akashi Y. Suzuki T. Hosokawa C. (2004). Differeces in receptor specificity of human and murine respiroviruses. Glycobiology 14, 1156–1156.

[B36] Tappert M. M. Porterfield J. Z. Mehta-D'Souza P. Gulati S. Air G. M. (2013). Quantitative comparison of human parainfluenza virus hemagglutinin-neuraminidase receptor binding and receptor cleavage. J. Virol. 87, 8962–8970. 10.1128/JVI.00739-13 23740997 PMC3754076

[B37] Tong J. Fu Y. Wu N. H. Rohde M. Meng F. Valentin-Weigand P.. (2018). Sialic acid-dependent interaction of group B streptococci with influenza virus-infected cells reveals a novel adherence and invasion mechanism. Cell. Microbiol. 20:e12818. 10.1111/cmi.12818 29272058

[B38] Van Calsteren M. R. Gagnon F. Lacouture S. Fittipaldi N. Gottschalk M. (2010). Structure determination of *Streptococcus suis* serotype 2 capsular polysaccharide. Biochem. Cell Biol. Biochim. Biolog. Cell. 88, 513–525. 10.1139/O09-170 20555393

[B39] Wang J. Y. Chen Z. L. Li C. S. Cao X. L. Wang R. Tang C.. (2015). The distribution of sialic acid receptors of avian influenza virus in the reproductive tract of laying hens. Mol. Cell. Probes 29, 129–134. 10.1016/j.mcp.2015.01.002 25725345

[B40] Willenborg J. Willms D. Bertram R. Goethe R. Valentin-Weigand P. (2014). Characterization of multi-drug tolerant persister cells in *Streptococcus suis*. BMC Microbiol. 14:120 10.1186/1471-2180-14-120 24885389 PMC4040513

