# Supplementary figures and images for: The Sialic Acid Binding Activity of Human Parainfluenza Virus 3 and Mumps Virus Glycoproteins Enhances the Adherence of Group B Streptococci to HEp-2 Cells
Source: Front Cell Infect Microbiol. 2018 Aug 17;8:280. doi: 10.3389/fcimb.2018.00280 (PMC6107845; doi:10.3389/fcimb.2018.00280)

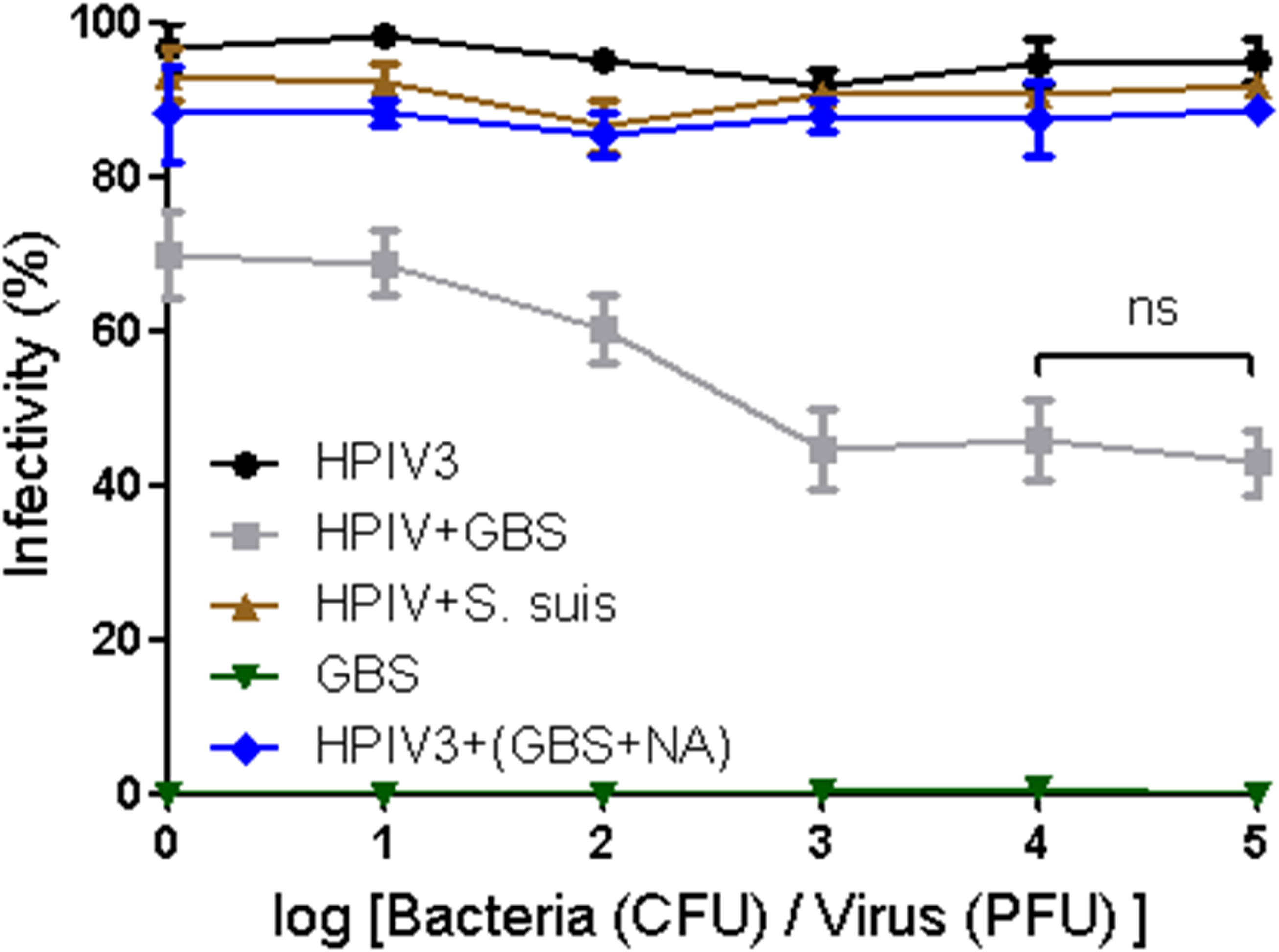

Supplement: Supplemental Figure 1 — Co-sedimention of HPIV3 with GBS or S. suis at different virus/bacteria ratios. Bacteria (108 CFU) were mixed with different amounts of infectious virus. After incubation of HPIV3 with GBS, NA-pretreated GBS or S. suis, the bacteria were pelleted by low-speed centrifugation. The supernatants were analyzed for viral infectivity by plaque titration on HEp-2 cells. Statistical significance was determined with one-way ANOVA, *P < 0.05. ns, not significant. [file Image_1.TIF]
